# [^18^F]NaF PET/CT to assess bone healing capacity in orthopaedic trauma surgery: a feasibility study

**DOI:** 10.1007/s00259-025-07209-y

**Published:** 2025-03-27

**Authors:** N. D. van Rijsewijk, M. Wouthuyzen-Bakker, J. H. van Snick, J. van Sluis, K. Ten Duis, A. W. J. M. Glaudemans, F. F. A. IJpma

**Affiliations:** 1 Department of Nuclear Medicine and Molecular Imaging, University of Groningen, University Medical Center Groningen, Groningen, The Netherlands; 2 Department of Medical Microbiology and Infection Prevention, University of Groningen, University Medical Center Groningen, Groningen, The Netherlands; 3 Department of Trauma Surgery, University of Groningen, University Medical Center Groningen, Groningen, The Netherlands

**Keywords:** [^18^F]NaF PET/CT, Bone healing, Non-union, Delayed union, Pseudoartrosis, Trauma surgery, Orthopaedic surgery

## Abstract

**Objective:**

Evaluation of bone healing potential in cases of delayed union or non-union remains a challenge. This study evaluated the use of [^18^F]NaF PET/CT to assess bone healing and its role in guiding clinical decision-making in orthopaedic trauma surgery.

**Methods:**

From June 2019 to December 2024, a prospective study was conducted in a level 1 trauma center. The study included 18 patients (20 fractures: 15 non-unions, 5 bone defects) all showing impaired bone healing after initial surgical fracture treatment, who were eligible for revision surgery. Dynamic (first 10 min post-tracer injection) and static (1-hour post-injection) [^18^F]NaF PET/CT scans were performed to assess bone healing capacity through qualitative and quantitative analysis. The relationship between scan results and treatment outcome was evaluated.

**Results:**

Increased [^18^F]NaF uptake, suggestive for bone healing capacity, was observed in all fractures (20/20). Based on the positive [^18^F]NaF PET/CT results, watchful waiting was recommended for all fractures, with two patients preferring surgery. After prolonged watchful waiting, 72% (13/18) showed bone healing progression. Notably, atrophic fractures displayed biological activity, and non-unions demonstrated sustained metabolic activity even beyond 12 months. Most fractures requiring revision surgery (80%, 4/5) needed additional stabilization. Ultimately, [^18^F]NaF PET/CT imaging helped to avoid revision surgery in 65% of all fractures (13/20). Uptake pattern analysis suggest that whole-bone uptake could potentially indicate surgical need (χ² = 5.62, *p* = 0.018). Quantitative assessment provided no additional information.

**Conclusion:**

[^18^F]NaF PET/CT imaging provides valuable insights into bone healing potential, especially in non-unions, helping guide clinical decisions and reducing unnecessary surgeries. This study challenges traditional non-union definitions by highlighting the biological activity of both atrophic and hypertrophic fractures.

## Introduction

Fractures are common injuries in orthopaedic trauma. Effective management involves alignment and stabilization to promote an optimal environment for bone healing. Fracture bone healing is a complex biological process, with the aim of reformation of bone structure and function. However, delayed union and non-union can occur despite fixation and is a frequent challenge in orthopaedic trauma surgery. Delayed union is typically defined as insufficient bone healing after a period of nine months after surgical fixation. Non-union, a more severe form of healing problem, occurs when the fracture does not show any healing potential. The definition of non-union is mainly time-based, without consensus, ranging from 3 to 12 months [1]. This condition can have personal and societal impacts, leading to persistent pain, swelling, limited range of motion, and delayed return to normal activities, including work [2]. Both conditions often lead to difficult clinical decisions regarding whether to wait for natural healing to progress or to intervene surgically.

Evaluation of bone healing potential in case of delayed union or non-union remains a challenge. Conventional imaging modalities such as radiographs and computed tomography (CT) scans are commonly used [3], but have limitations, particularly in detecting minor signs of bone healing, such as metabolic or soft tissue changes. Bone scintigraphy, a conventional metabolic imaging method using technetium-99m diphosphonates ([^99m^Tc]MDP, [^99m^Tc]HDP, [^99m^Tc]HDMP), detects areas of increased bone metabolism by binding to the organic bone matrix. Uptake reflects not just mineralization but also bone vascularity and turnover, potentially visualizing both osteoblastic and osteoclastic activity indirectly. Therefore, this imaging modality can be used to assess bone healing but may lack precision; increased uptake cannot only occur in cases of fracture healing but also in a variety of other conditions such as soft tissue injuries, recent operations, and infections [4, 5]. Furthermore, the spatial resolution and possibilities for quantification using this technique are limited. Given the inadequacies of these imaging techniques in the evaluation of bone healing, more sensitive and specific modalities are needed.

^18^F-Sodium fluoride positron emission tomography ([^18^F]NaF PET) has appeared as a promising imaging technique in this context. [^18^F]NaF PET/CT measures the uptake of fluoride ions, which integrate into the bone matrix during the process of mineralization during bone healing, thus reflecting areas of active bone formation as a direct marker [4]. This radiotracer shows high sensitivity and specificity in detecting metabolic changes associated with bone healing; in comparison to bone scintigraphy, [^18^F]NaF has a higher affinity for hydroxyapatite and has a lower background signal due to faster clearance. Furthermore, [^18^F]NaF PET allows better resolution and quantitative measurements more easily, providing objective data on bone metabolism. This quantitative aspect might be helpful to differentiate between normal and pathological processes. Therefore, [^18^F]NaF PET/CT has the potential to enhance the clinician’s ability to assess bone healing and determine whether fractures are progressing toward union or if surgical intervention may be necessary.

Despite its potential, [^18^F]NaF PET/CT is not routinely used in clinical practice in the context of evaluation of bone healing after orthopaedic trauma surgery, and there is limited research assessing its helpfulness. At the moment, there is a knowledge-gap regarding the clinical impact of [^18^F]NaF PET/CT and whether semi-quantitative measurements can effectively guide decision-making in cases of complex bone healing.

The aim of this study is to evaluate the use of [^18^F]NaF PET/CT in assessing bone healing in patients after orthopaedic trauma surgery, and whether semi-quantitative measurements obtained from [^18^F]NaF PET/CT are helpful in diagnosing delayed healing and guiding treatment decisions. Our hypothesis is that [^18^F]NaF PET/CT can provide an accurate assessment of bone healing capacity in patients with impaired bone healing and can differentiate between patients who achieve bone healing within an extended expectant approach and those requiring revision surgery. Therefore, the research questions in this study were: (1) what is the impact of [^18^F]NaF PET/CT imaging on clinical decision-making in the management of patients referred for bone healing assessment after surgical fracture treatment? and subordinate question (2) what is the relationship between quantification of [^18^F]NaF PET/CT and bone healing potential?

## Materials and methods

### Study population

Patients referred to the nuclear medicine department by trauma or orthopaedic surgeons for [^18^F]NaF PET/CT assessment of bone healing processes in the upper and lower extremities after initial surgical fracture treatment were included in this single center prospective study. Patient inclusion was conducted in the University Medical Center Groningen, a tertiary (level 1) trauma center, between June 2019 and December 2024. Included were patients with impaired bone healing after initial surgical fracture treatment for non-union or bone defects, all of whom were eligible for revision surgery. Non-union was defined according to the FDA criteria, which states that the fracture is not healed after standard treatment within the first 9 months after injury and shows no signs of healing progression for at least 3 months on conventional imaging (radiographs, CT, MRI) [1]. Follow-up was achieved for at least 6 months after [^18^F]NaF imaging. Patients were excluded when concurrent oncologic bone processes were expected. The local Medical Ethical Commission approved this prospective study (METC number 2019/00207).

For all included patients, data on gender, age, weight, injected tracer activity, used PET/CT scanner, possible confounders (prior chemo- or radiotherapy, use of bisphosphonates), fracture site, clinical symptoms, history of fracture-related infections, surgery dates, prior imaging modalities, treatment, follow-up decisions and follow-up duration were acquired by using their electronic patient records.

### PET/CT acquisition and image analysis

The acquisition protocol consisted of a dynamic scan performed in the first ten minutes immediately after tracer injection using only one fixed bed position (26.5 cm in Biograph Vision, 106 cm in Biograph Vision Quadra), with the fracture itself as the main focus. This was followed by a static scan within a time-interval of 60 ± 5 min after intravenous injection of [^18^F]NaF according to existing clinical camera protocols. Static [^18^F]NaF PET/CT scans were mostly performed with a total body acquisition, using two different PET/CT systems (Biograph Vision and Biograph Vision Quadra, Siemens Healthineers, Knoxville, USA). Therefore, the existing clinical camera protocols were used (mostly using continuous bed motion with an average speed of 1.6 millimeters per second, or 3 min per bed position). The scans were reconstructed using EARL2 compliant settings: Poisson ordered-subset expectation maximization (OP-OSEM) 3-dimensional (3D) iterative algorithm with 4 iterations and 5 subsets, with application of time of flight (ToF), resolution modeling (PSF) and a Gaussian filter of 5 mm, using a matrix size of 220 × 220 and voxel size of 3,3 × 3,3 × 2,0 mm. From June 2019 through December 2022 patients received a standard intravenous administration of 3 MBq per kilogram body of [^18^F]NaF, from January 2023 onwards patients received a standard intravenous administration of 2 MBq per kilogram body weight. An additional low-dose CT was obtained for attenuation correction.

[^18^F]NaF PET/CT images were assessed both qualitatively and semi-quantitatively. The qualitative assessment was based on a visual grading scale and was performed at the fracture location. The visual 4-point grading scale classified the [^18^F]NaF uptake: grade 0 - no [^18^F]NaF uptake; grade 1– [^18^F]NaF uptake less than the axial skeleton, grade 2– [^18^F]NaF uptake comparable to the axial skeleton; grade 3– [^18^F]NaF uptake exceeds the uptake in the axial skeleton. A grade 2 or 3 [^18^F]NaF uptake at the fracture location was considered positive for ongoing bone healing capacity on [^18^F]NaF PET/CT. Additionally, the uptake pattern was evaluated to distinguish between focal and whole-bone uptake, with whole-bone uptake defined as grade 2 or 3 uptake involving more than 75% of the bone.

Semi-quantitative analysis was performed on static and dynamic reconstructed images using standardized uptake values according to the EANM procedure guidelines [6]. Hence, ellipsoidal volumes of interest were used to measure the SUV_peak_ at the fracture site. SUV_mean_ measurements were performed in the axial skeleton (lumbar spine, region L1-L5) and the contralateral side of the fracture using a threshold of 40% of SUV_max_ to delineate the bone uptake, in order to quantify physiological [^18^F]NaF uptake. Soft tissue (muscle tissue, gluteus medius) uptake was measured using a spherical volume of interest. Time activity curves were created for the dynamic reconstructions, using twenty frames in the following order: 7 × 10 s, 8 × 30 s and 5 × 1 min. Therefore, an ellipsoidal VOI was created at the fracture site and contralateral side, avoiding the greater vasculature. All measurements were performed by a physician researcher (NvR) using Syngo.via VB60 software (Siemens Healthineers, Knoxville, USA). Uncertainties were discussed and solved by consensus with an experienced nuclear medicine physician (AG).

### Outcome measures

The primary outcome measure of this study is the impact of [^18^F]NaF PET/CT imaging on clinical decision-making in the management of patients referred for bone healing assessment after initial trauma surgery. Specifically, the study evaluates whether [^18^F]NaF PET/CT findings indicating ongoing bone healing potential can support the continuation of expectant management instead of proceeding with revision surgery. The assessment focuses on whether patients managed expectantly based on [^18^F]NaF PET/CT results ultimately achieved successful fracture healing (monitored by radiographs or CT-scans, and clinical follow-up), thereby preventing unnecessary surgical interventions.

Additionally, the study examines cases where surgery was considered necessary either due to clinical indications or at the patient’s request. This includes scenarios where physicians recommended an extended watchful waiting approach based on [^18^F]NaF PET/CT findings, but patients opted for revision surgery with the expectation of more rapid clinical recovery. The outcomes of these decisions were analyzed by descriptive statistics to understand the role of patient preferences and clinical judgment in the management of bone healing post-initial trauma surgery.

### Statistical analysis

Descriptive statistics were used to provide an overview of patient’s characteristics and quantitative assessment. Given the small sample size, non-parametric tests were utilized to ensure the robustness of the statistical analysis [7]. A chi-squared test of independence was used to evaluate the association between the [^18^F]NaF uptake pattern and clinical outcomes after the expectant approach. Fisher’s Exact Tests were used to assess the association between the visual [^18^F]NaF uptake intensity and pattern, and the time span for spontaneous fracture healing. For quantitative assessment comparisons were performed to identify significant differences in SUV and ratios between the group of patients in which bone healing occurred during the expectant approach and the group of patients who still required surgery after the expectant approach using the Mann-Whitney U test. Spearman’s rank correlation was used to examine the relationship between the time span for spontaneous fracture healing and fracture SUV_peak_ values. Comparison of the time activity curves at the fracture site, the contralateral side and their ratio between groups was performed using the mixed-effects model.

All results were considered statistically significant with a *p*-value < 0.05. Missing data were excluded pairwise. Statistical analyses were performed using IBM^®^ SPSS^®^ Statistics (Version 28; IBM Corp., Armonk, NY, USA) and Python (version 3.12) with the following packages: *numpy*, *pandas*, *matplotlib*, *statsmodels* and *seaborn*.

## Results

### Patients and fractures

Eighteen patients (13 males, 5 females) were included in this study. Within this cohort, a total of twenty fractures were analyzed. The median [IQR] age of the patients at time of [^18^F]NaF PET/CT was 42.0 [32.2–48.8] years. Their median [IQR] body mass index was 26.2 [24.2–29.0]. 50% of all patients were smokers. Two patients (11%) were diagnosed with osteoporosis. The indication for performing [^18^F]NaF PET/CT was non-union in fifteen fractures and monitoring bone healing after bone defect surgery in five fractures. These procedures were performed for large bone defects, and consisted of: cancellous bone graft in two patients, one segmental bone transport, one bone allograft combined with plating, and one vascularized fibula autograft. Fracture locations were femur (50%), tibia (30%) and humerus (20%).

The median [IQR] time between trauma and [^18^F]NaF PET/CT imaging was 21.1 [16.6–42.0] months. The median [IQR] time between the last surgery at the fracture site and [^18^F]NaF PET/CT imaging was 13.7 [10.2–18.4] months. The median [IQR] follow-up time after [^18^F]NaF PET/CT is 12.3 [9.1–21.3] months. The extended watchful waiting period in all fractures lasted for a median [IQR] of 11.7 [8.9–13.8] months, and the total watchful waiting period since last surgical intervention for 25.9 [20.2–30.7] months.

Characteristics are summarized in Table 1.

**Table 1:** Summary of patient, fracture and scan characteristics. Continuous data is presented as median [IQR]. Abbreviations: y = years, m = months

Characteristic	Value	
**Patients**	**18**	
Gender		
Male	13	(72%)
Female	5	(28%)
Age (y)	42.0	[32.2–48.8]
BMI	26.2	[24.2–29.0]
20–25	5	(28%)
25–30	10	(55%)
> 30	3	(17%)
Smoking		
Yes	8	(45%)
No	10	(55%)
Osteoporosis	2	(11%)
ASA - score		
1	7	(39%)
2	10	(55%)
3	1	(6%)
**Trauma and fractures**	**20**	
Trauma mechanism		
High-energy trauma	15	(75%)
Low-energy trauma	5	(25%)
History of treated fracture-related infection	9	(45%)
Fracture location		
Femur	10	(50%)
Tibia	6	(30%)
Humerus	4	(20%)
Initial treatment		
Nail	11	(55%)
Plate	9	(45%)
**[** ^**18**^ **F]NaF PET/CT imaging**	**20**	
[^18^F]NaF PET/CT imaging indications		
Non-union	15	(75%)
Monitoring bone healing after bone defect surgery	5	(25%)
Time between trauma and [^18^F]NaF imaging (m)	21.1	[16.6–42.0]
Time between last surgery and [^18^F]NaF imaging (m)	13.7	[10.2–18.4]
Follow-up time after [^18^F]NaF imaging (m)	12.3	[9.1–21.3]
Time from [^18^F]NaF imaging to additional surgery decision (m)	15.0	[10.0–17.3]
Time from [^18^F]NaF imaging to spontaneous healing (m)	11.2	[7.7–12.5]
Time from trauma to spontaneous healing (m)	32.8	[23.8–53.1]
Time from last surgery to spontaneous healing (m)	23.9	[20.2–30.7]

### Clinical decision-making

After performing [^18^F]NaF PET/CT, all fractures were considered to show osteoblastic activity and expected to have bone healing potential (≥ grade 2). Therefore, in 20 out of 20 (100%) fractures an extended period of watchful waiting was suggested by the treating physician.

Within the group of fifteen non-unions, two patients still opted for surgery despite that there was osteoblastic activity visualized on the [^18^F]NaF PET/CT. An extended watchful waiting period was opted in the other thirteen non-unions. Nine out of thirteen (69%) fractures healed spontaneously during this extended period of watchful waiting. Five of these spontaneously healed fractures were atrophic and four hypertrophic. However, during the follow-up period after [^18^F]NaF PET/CT, four of thirteen (31%) fractures required surgery to achieve bone healing. Two of those (both hypertrophic) needed plate augmentation on top of their initial pen osteosynthesis to improve the stability of the fracture. One patient experienced failure of the osteosynthesis material and therefore needed surgery. The last patient suffered from a low-grade fracture-related infection and needed to be treated by debridement, antibiotics and implant retention.

In the patients who underwent [^18^F]NaF PET/CT for monitoring bone healing after surgical treatment of a bone defect, four fractures (80%) healed spontaneously during extended watchful waiting and only in one patient (20%) the physician suggested surgery due to implant failure associated with a fibular allograft. This single patient was known with osteoporosis and received medical treatment for this condition.

**Fig. 1:**
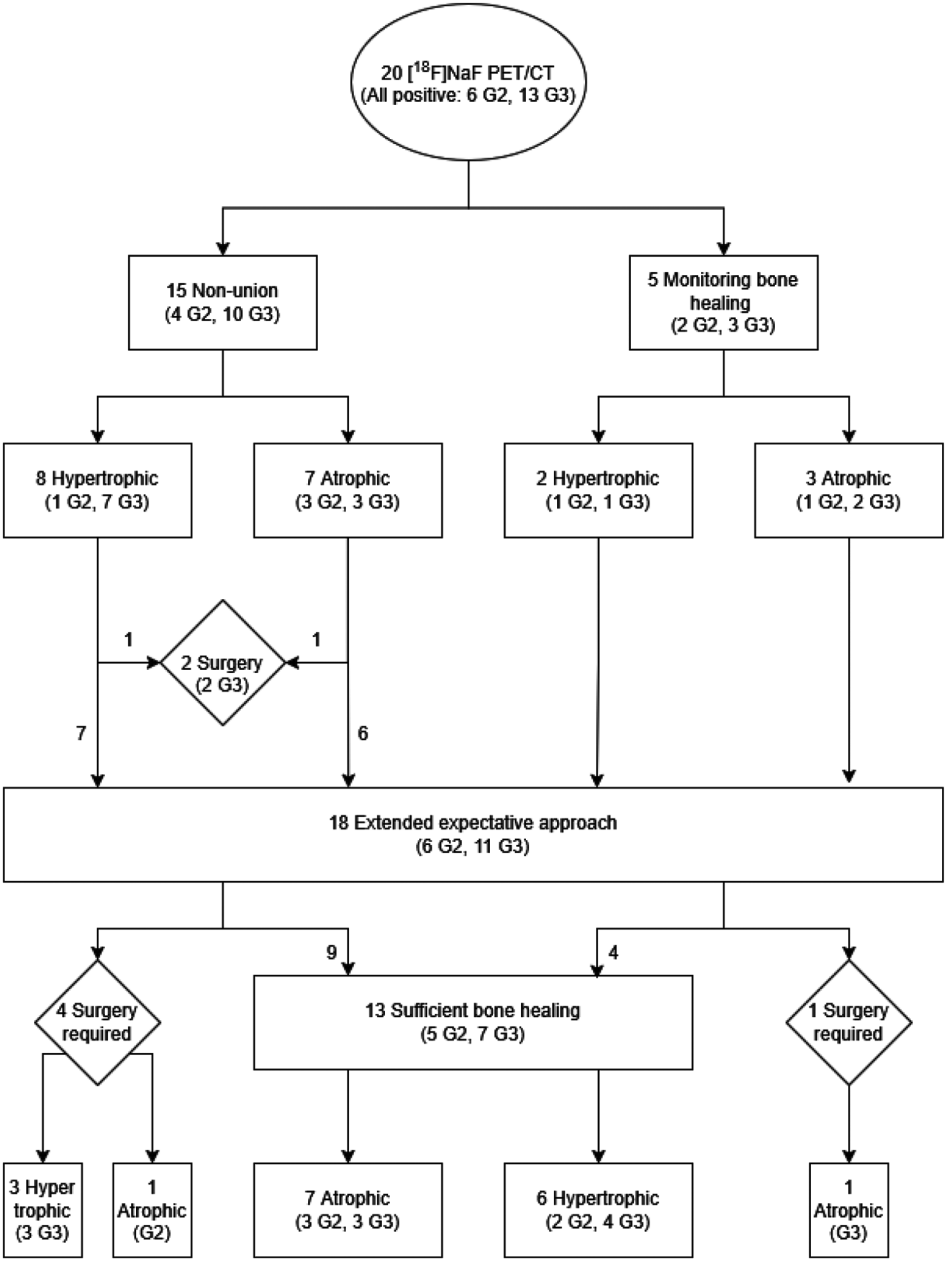
Flow diagram illustrating the referral and outcomes of 20 investigated fractures with [^18^F]NaF PET/CT for bone healing potential. Visual grading score is abbreviated as ‘G0’ to ‘G3’. There was one patient in which no grade could be assigned

**Fig. 2:**
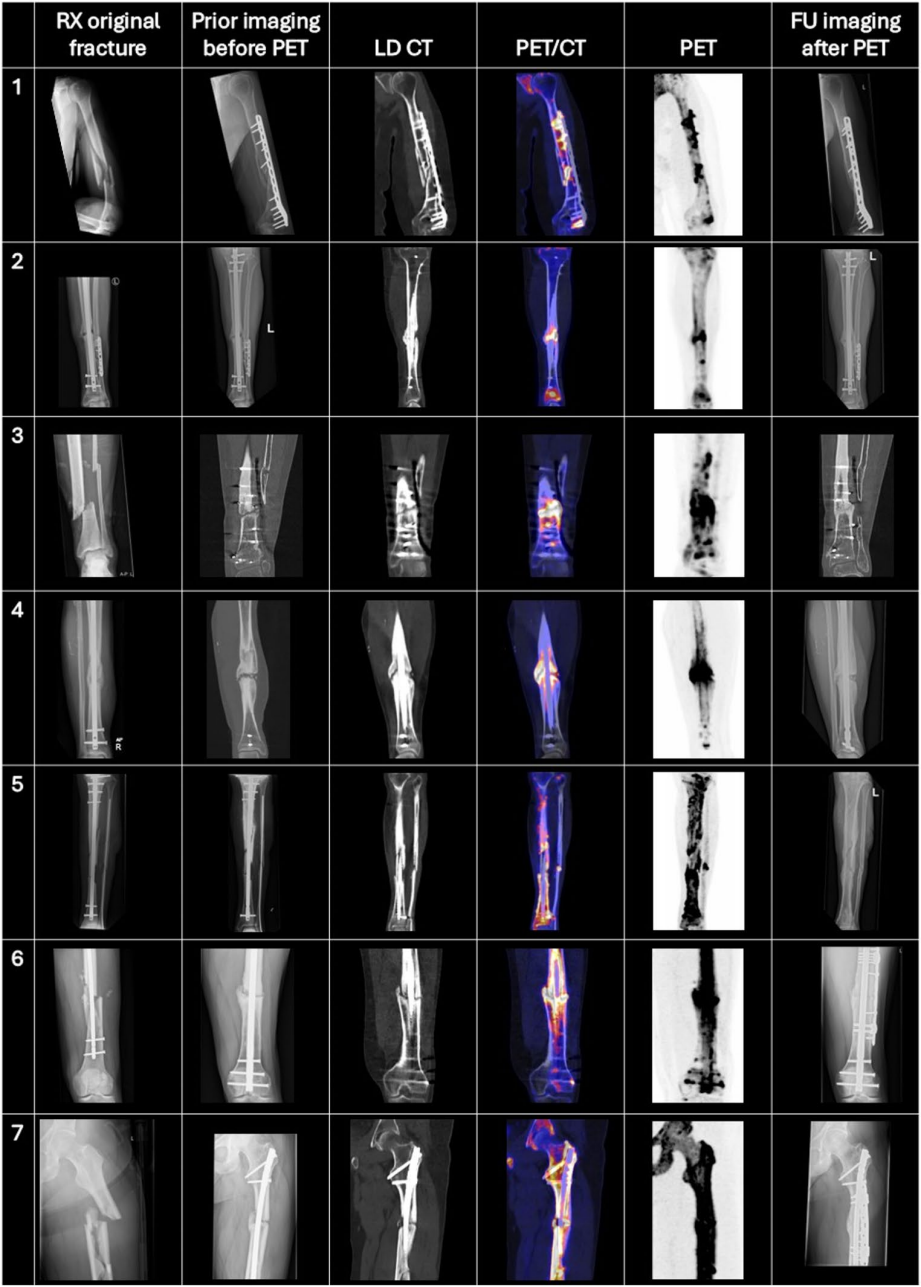
An illustration of sequential imaging in seven cases, progressing from the original fracture through prior imaging to [^18^F]NaF PET/CT and follow-up imaging. PET and fusion images do have a scale of 0 to 10 SUV. All patients show significant [^18^F]NaF uptake along the non-union site. Patient 1 to 4 show progression in fracture healing in the follow-up imaging during extended watchful waiting, so without any intervention. Patient 5 also shows fracture healing progression, however needed to have debridement, antibiotics and implant retention for a fracture-related infection during the extended period of watchful waiting. No other bone healing enhancement treatments were performed in this patient. Patient 6 and 7 showed an increased whole-bone uptake pattern. Both patients required treatment with plate augmentation to address fracture instability and achieve bone healing at the non-union site. Abbreviations: RX = radiography, LD = low dose, FU = follow = up

**Fig. 3:**
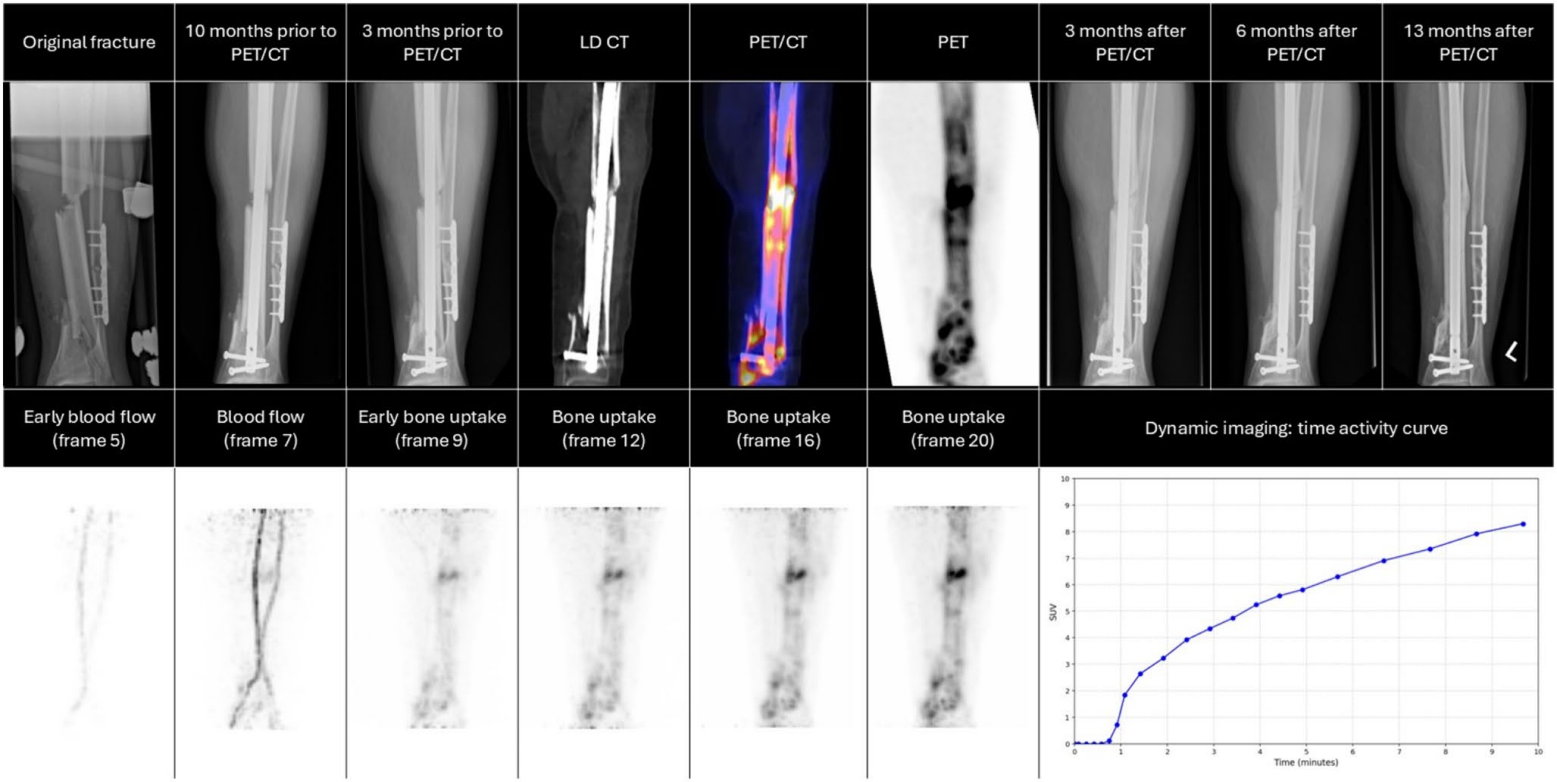
Example of a patient. The first row illustrates the progression of bone healing of the original fracture (14 months prior to PET/CT with minimal or no signs of fracture healing observed on radiographs between 10 and 3 months prior to PET/CT imaging. [^18^F]NaF PET/CT was positive (grade 3), indicating potential for bone healing. Three months after PET/CT imaging, radiographs showed signs of healing progression, which continued over time. By 6 months, further healing was evident,> and full consolidation was achieved at 13 months. The second row displays individual frames from the first 10 min of post-injection dynamic imaging, highlighting [^18^F]NaF uptake at the fracture site. The final section presents the time-activity curve for the fracture, demonstrating a gradual rise in [^18^F]NaF uptake over time

In total, 13/18 (72%) fractures spontaneously healed during extended watchful waiting, taking a median [IQR] time of 11.2 [7.7–12.5] months from [^18^F]NaF PET/CT. A total of 5 (28%) patients required revision surgery, taking a median [IQR] time of 15.0 [10.0–17.3] months from [^18^F]NaF imaging to additional surgery decision. Two patients preferred surgery over an extended expectant approach. Therefore, revision surgeries could be avoided in 65% of all fractures (13/20) by performing [^18^F]NaF PET/CT to evaluate bone healing potential in this study. A summary can be found in Fig. 1 and an illustration of cases in Figs. 2 and 3.

### Scans and qualitative measurements

A total of twenty fractures were scanned in nineteen unique scans (13 Vision, 7 Quadra). In sixteen patients a first ten-minute dynamic acquisition was acquired and could be analyzed (10 Vision, 6 Quadra). Median [IQR] administered radiation dose was 212.5 [172.5- 242.5] MBq. All fractures showed high [^18^F]NaF uptake, of which thirteen fractures showed a grade 3 uptake and 6 fractures a grade 2 uptake. In one patient no axial skeleton was visualized, so no grade could be assigned. No fractures scored grade 0 or 1 uptake. Patients requiring revision surgery for fracture healing after the extended expectant follow-up period showed mainly grade 3 uptake (4/5, 80%). No significant association was found between the visual [^18^F]NaF uptake intensity or pattern, and the time span for fracture healing (*p* = 0.576 and *p* = 1.000, respectively). An overview of visual grading scores can be found in Fig. 1.

Five patients demonstrated increased whole-bone uptake by uptake pattern analysis. Of these, four patients required surgery, while one patient with increased whole-bone uptake healed spontaneously. In contrast, 11 patients with increased focal uptake did not require surgery. A chi-squared test of independence demonstrated a significant association between the increased whole bone uptake pattern and the need for surgery (χ² = 5.62, *p* = 0.018).

### Quantitative measurements

**Fig. 4:**
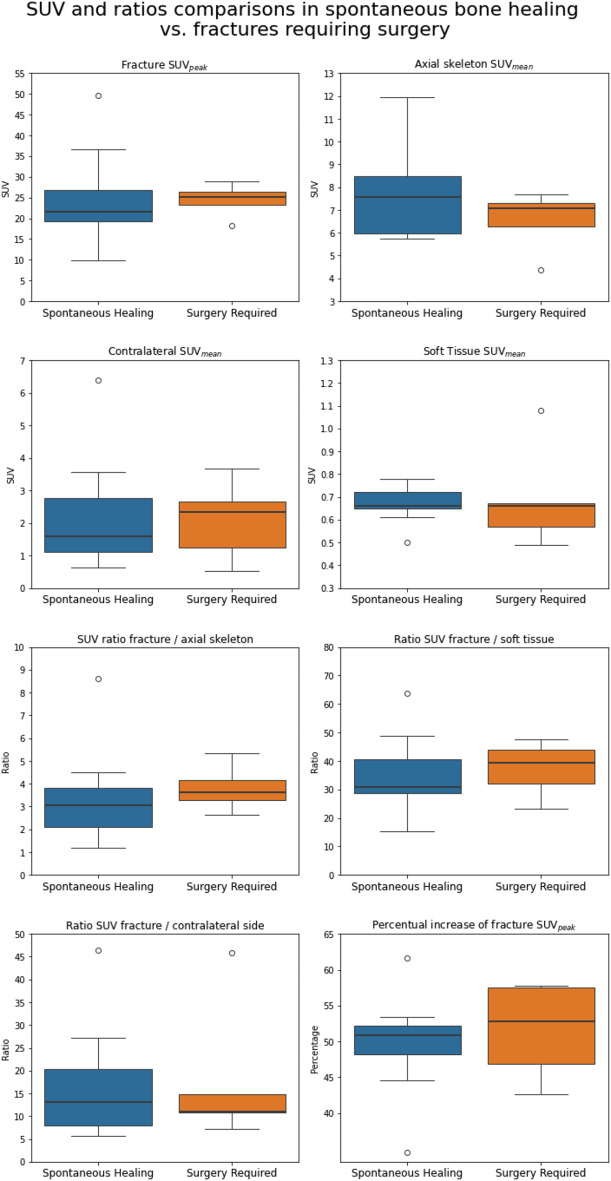
Comparison of Standard Uptake Values (SUV) between patients with spontaneous bone healing during an extended expentant period (blue) and those requiring revision surgery after an extended expectant approach (orange). Boxplots depict the median and interquartile range (IQR) for the measurements of SUV_peak_ at the fracture location and the SUV_mean_ at the contralateral side, SUV_mean_ in the axial skeleton, and SUV_mean_ in soft tissue, their ratios and the percentual increase in SUV_peak_ at the fracture site one hour post-injection relative to the SUV_peak_ measured between minutes 9 and 10 post-injection. Outliers are represented as individual points beyond the whiskers, which denote 1.5 times the IQR. The boxplots demonstrate no significant differences in all SUV measurements and their ratios between patients with spontaneous healing and those requiring surgery. Across all regions of interest, the SUV measurements and their ratios show considerable overlap between the two groups in the medians and interquartile ranges. These findings suggest similar tracer uptake patterns, with no clear separation between the two groups based on SUV values

The results of the quantitative measurements are summarized in Table 2, presenting the overall group statistics and separate statistics for the group of spontaneous bone healing and the group who needed revision surgery after an extended period of watchful waiting. There are no significant differences between the two groups in SUV measurements, ratio calculations and percentual change. Comparisons between the two groups are also visualized in Fig. 4.

**Table 2:** Summary of quantitative measurements. All statistics are presented as median [IQR]

	All fractures (*N* = 20)		Spontaneous healed fractures (*N* = 13)		Fractures requiring surgery (*N* = 5)		*p*
**SUV measurements**							
Fracture SUV_peak_	23.09	[18.78–27.91]	21.66	[19.26–26.81]	25.09	[23.35–26.45]	*0.522*
Contralateral SUV_mean_	1.72	[1.14–2.84]	1.60	[1.07–2.95]	2.33	[1.24–2.65]	*0.833*
Axial skeleton SUV_mean_	7.19	[5.98–7.70]	7.58	[5.98–8.47]	7.06	[5.65–7.45]	*0.327*
Soft tissue SUV_mean_	0.67	[0.65–0.76]	0.66	[0.65–0.72]	0.66	[0.57–0.67]	*0.586*
**Ratio**							
Fracture / Contralateral side	10.94	[8.87–17.62]	13.02	[7.79–20.97]	10.94	[10.77–14.75]	*0.916*
Fracture / Axial skeleton	3.41	[2.36–4.17]	3.07	[2.10–3.82]	3.63	[3.06–4.55]	*0.296*
Fracture / Soft tissue	31.50	[27.72–42.28]	30.91	[28.67–40.62]	39.48	[32.09–43.94]	*0.657*
**Percentual change**							
Last fracture SUV_peak_ dynamic to SUV_peak_ static	50.55	[47.89–52.90]	50.82	[47.98–52.36]	52.85	[45.48–57.59]	*0.777*

### Dynamic imaging

**Fig. 5:**
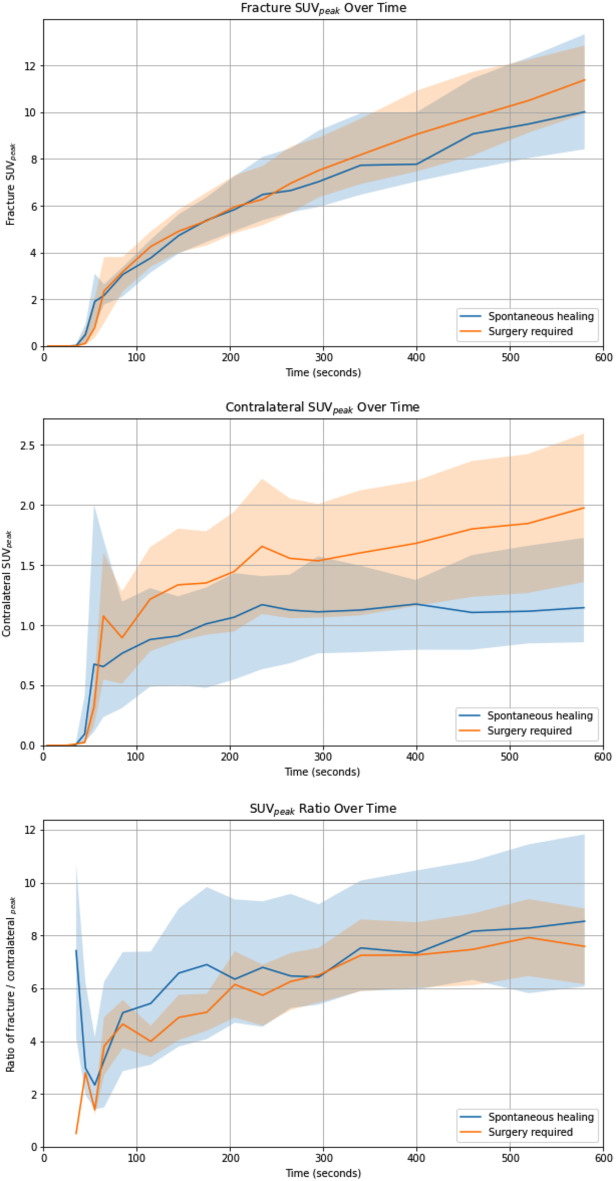
SUV_peak_ measurements over time at the fracture site, contralateral regions and their ratios in groups with patients with successful extended watchful waiting and patients requiring revision surgery for bone healing. These graphs illustrate the progression of the SUV_peak_ values over time in fractured bone, contralateral regions and their ratios for two patient groups: patients with successful expectant fracture treatment (blue line) and patients requiring revision surgery for bone healing (orange line). Data points show median SUV_peak_ values at various time intervals (7 × 10s, 8 × 30s and 5 × 1 min), and the shaded regions represent the interquartile ranges for each group. The first graph, comparing SUV_peak_ over time at the fracture site, shows a steady increase in SUV_peak_ over time for both groups, with no significant difference. The second graph, shows a gradual rise in SUV_peak_ in the patient group requiring surgery (orange). The group with spontaneously healed fractures during an extended expectant approach (blue) remains relatively stable over time. This finding suggests differences in blood flow, metabolic activity or uptake in the contralateral regions. However, this difference is not significant (*p* = 0.191). In the last graph, both groups exhibit a rising trend in SUV_peak_ ratio (fracture/contralateral side), with the group of patients requiring surgery showing higher variability but no significant difference between the groups

Time-activity curves of [^18^F]NaF in the fracture and contralateral side and their ratio are shown in Fig. 5. In this study, SUV_peak_ measurements were compared between two groups: those with fractures that healed spontaneously under expectant treatment (blue) and those requiring surgical intervention (orange). SUV_peak_ of the fracture increased over time for both groups. In the fractured bone regions, both groups showed a sharp rise in SUV_peak_ within the first 100 s, followed by a more gradual increase. By the end of the observation period, the surgically treated group showed a slightly higher SUV_peak_, though the overlap in interquartile ranges suggests no statistically significant difference (*β* = -0.002, *p* = 0.657). In the contralateral (non-fractured) bone regions, the group that did require revision surgery showed a more pronounced rise in SUV_peak_, while the expectant management group remained relatively stable after the initial uptake. However, the difference between the two groups is not significant (*β* = -0.001, *p* = 0.191). The SUV_peak_ ratio curves of the fractured regions over the contralateral regions showed a steady increase in both groups, with no significant differences (*β* = -0.007, *p* = 0.994). The variability of the SUV_peak_ ratio in the surgically treated group was notably higher.

## Discussion

This study presents a novel approach to evaluate bone healing potential in cases of impaired bone healing following orthopaedic trauma surgery by using [^18^F]NaF PET/CT imaging. While traditional methods for assessing bone healing primarily rely on clinical evaluation and radiographic imaging, the use of [^18^F]NaF PET/CT offers a visual, dynamic and quantitative assessment of bone metabolic activity, providing insights into the healing process that may not be apparent through standard imaging. Our findings demonstrate [^18^F]NaF uptake in all studied fractures with impaired bone healing, leading to the suggestion of an extended period of watchful waiting. Two patients opted for surgery. Thirteen out of eighteen (72%) remaining fractures, showed bone healing progression in the extended expectant approach. This resulted in avoiding revision surgery in 65% of all fractures. [^18^F]NaF PET/CT showed healing potential in both hyper- and atrophic fractures, with 69% of non-unions showing significant healing without the need for surgical intervention, challenging the current definition of non-unions, which associate atrophic types with reduced biological activity and rely on a time-based criterion of nine months without healing.

Our study shows that patients diagnosed with non-union still exhibit increased [^18^F]NaF uptake at the fracture site, suggesting ongoing osteoblastic activity. This implies that active bone healing continues for a long time, even in those classified as non-unions. These findings challenge the time-based definition of non-union and indicate that many of these fractures still have the potential for healing if managed expectantly. Specifically, this study found that 69% of non-unions healed even after 12 months since the last surgical intervention. This finding demonstrates that a significant number of fractures show ongoing bone activity beyond the traditional time frame and supports the use of [^18^F]NaF PET/CT for bone healing assessment.

The clinical impact of [^18^F]NaF PET/CT is significant, as it can help avoid unnecessary surgeries in fracture management, as demonstrated in this study (65% of cases). By providing detailed information on osteoblastic activity, [^18^F]NaF PET/CT can guide clinicians in deciding whether revision surgery is primarily needed or an extended expectant approach might be sufficient. Importantly, patient factors such as pain, functional limitations, and overall health must also be considered, as these may affect the feasibility of an extended expectant management. As such, [^18^F]NaF PET/CT should be used in conjunction with a comprehensive clinical assessment for individualized fracture evaluation, particularly in impaired healing scenarios. This imaging modality may guide decisions for an extended expectant approach, as high uptake in non-unions suggests the possibility of spontaneous bone healing.

Unfortunately, quantification of both static and dynamic imaging did not provide additional information and could not distinguish those patients in which surgery was still required after a prolonged expectant treatment period. However, the results of uptake pattern analysis suggest that whole-bone uptake could potentially be an indicator for the need of surgery (χ² = 5.62, *p* = 0.018). In this study, 31% of non-union fractures required surgery after an extended period of watchful waiting, highlighting that expectant approaches alone are not always sufficient. In 75% of these cases, the need for surgery was due to instability of the fracture, which failed to provide the necessary support for the healing bone, leading to the need for surgical revision. Fracture instability significantly impacts the bone healing process, as it can prevent proper alignment and delay healing. While [^18^F]NaF PET/CT is valuable for assessing bone metabolism and identifying areas of active healing, it does not provide direct information about the biomechanical stability of the fracture site. Nevertheless, our findings indicates that fractures with instability may exhibit a more widespread uptake pattern on [^18^F]NaF PET/CT, possibly reflecting compromised biomechanical stability at the fracture site. Movement of the osteosynthesis material due to instability can cause repetitive (micro)damage to the bone, leading to more extensive remodeling across a larger portion of the bone rather than being localized. This increased remodeling could be reflected as increased [¹⁸F]NaF uptake, further supporting the association between instability and the need for surgical intervention.

Radiographic scores for predicting non-unions were invented earlier, and showed promising results. They are based on the radiopaque visibility of the formation of callus and mostly applied within the first three months [8, 9]. It is often thought that atrophic non-unions are associated with reduced biological activity and that hypertrophic non-unions relate more to insufficient stability of the surgical fixation [10]. However, our [^18^F]NaF PET scans indicate that both types of non-unions can show considerable bone activity even after about one year. More specifically, 78% (7/9) of atrophic fractures spontaneously healed during the extended period of watchful waiting. The majority of fractures still requiring surgery needed stabilization (80%). Fracture healing progressed after surgical intervention, both in hyper- and atrophic fractures. Therefore, we believe that most non-unions, whether atrophic or hypertrophic, maintain sufficient biological activity, emphasizing the importance of stable operative fixation. In our study, based on our findings, we conclude that [^18^F]NaF PET is a valuable tool for assessing bone healing capacity in orthopaedic trauma surgery, potentially preventing unnecessary revision surgeries and allowing more time for natural bone healing if the fracture fixation is stable.

This study has some strengths and limitations. To our knowledge, it is the first to use [^18^F]NaF PET/CT to assess bone healing in non-union fractures, providing a proof-of-principle for this innovative imaging technique despite the small sample size. The study has significant clinical impact by challenging the time-based definition of non-union, and allowing for more personalized treatment by guiding decisions on whether to continue watchful waiting or opt for surgery. By monitoring bone activity over time, it helps avoid unnecessary revision surgeries (65% in this study), reducing both costs and patient risks. However, no cases in this study demonstrated an absence of increased [^18^F]NaF uptake at the fracture site, making it difficult to evaluate the negative predictive value of this imaging modality in predicting non-union. Additionally, the heterogeneity of the patient group, including those treated for bone defects with varying strategies, could affect the consistency of the findings; nonetheless, excluding these patients did not alter the results. Another limitation is the intrinsic inability of [^18^F]NaF PET/CT to evaluate the biomechanical status, which is needed for understanding the stability of the fracture site. Fracture stability is one of the key determinants in bone healing [11, 12], and without this information, clinicians may miss critical factors that influence the success of extended expectant management.

Future research should focus on increasing the number of studied fractures and longitudinal studies on bone healing of orthopaedic trauma surgery patients from trauma date to at least 12 months after fracture treatment. Hopefully, this could lead to a better understanding of fracture healing and help refine decision-making in orthopaedic trauma cases. However, for broader clinical implementation, challenges related to reimbursement must be addressed, as [¹⁸F]NaF PET/CT is not currently reimbursed in all European countries. Multicenter prospective studies are needed to support the integration of this technique into clinical guidelines and to secure appropriate reimbursement, enabling wider application of this promising imaging modality.

## Conclusion

[^18^F]NaF PET/CT imaging may provide valuable insights about the bone healing potential in cases with impaired fracture healing after orthopaedic trauma surgery. After performing [^18^F]NaF PET/CT, revision surgery was avoided in 65% of all patients. Furthermore, both hyper- and atrophic fractures showed bone healing potential. In our study, 69% of the non-union fractures still healed spontaneously after extended period of watchful waiting. Atrophic fractures were found to exhibit biological activity, and non-union fractures showed sustained activity beyond 12 months, both challenging traditional concepts in non-union diagnosis. High uptake on [^18^F]NaF PET/CT imaging, combined with adequate osteosynthesis stability, supports an extended expectant approach.

## Data Availability

The anonymized datasets generated during and/or analyzed during the current study are available from the corresponding author on reasonable request.
